# Alcohol, Athletic Performance and Recovery

**DOI:** 10.3390/nu2080781

**Published:** 2010-07-27

**Authors:** Luke D. Vella, David Cameron-Smith

**Affiliations:** Molecular Nutrition Unit, School of Exercise and Nutrition Sciences, Deakin University, Burwood, Victoria, 3125, Australia; Email: lve@deakin.edu.au

**Keywords:** ethanol, skeletal muscle, glycogen, protein synthesis

## Abstract

Alcohol consumption within elite sport has been continually reported both anecdotally within the media and quantitatively in the literature. The detrimental effects of alcohol on human physiology have been well documented, adversely influencing neural function, metabolism, cardiovascular physiology, thermoregulation and skeletal muscle myopathy. Remarkably, the downstream effects of alcohol consumption on exercise performance and recovery, has received less attention and as such is not well understood. The focus of this review is to identify the acute effects of alcohol on exercise performance and give a brief insight into explanatory factors.

## 1. Introduction

Athletes, like the rest of the population, consume alcohol. Sporting clubs and associations are frequently reported in the media to place bans or restrictions on the availability and consumption of alcohol by contracted athletes. Yet the same media organizations also report on alcohol-fuelled violence or misdemeanors perpetrated by these same athletes, suggesting anecdotally that athletes consume alcohol, occasionally to excess. This is quantitatively supported by dietary surveys of athletic populations that demonstrate self-reported alcohol intake constitutes up to 5% of the total daily energy intake in elite athletes [1]. However this is far from universal, as survey data reports either greater [2,3], or reduced [4,5] alcohol ingestion in athletic populations than the general community. This high variability in reported alcohol intake within athletic groups may in part be due to the characteristics of each sporting discipline. Alcohol intake appears to be positively associated with team sports where alcohol consumption is often encouraged as a component of team/group bonding and can be related to stress relief [6].

The detrimental effects of alcohol on human physiology have been well documented with acute alcohol ingestion affecting many aspects of metabolism, neural function, cardiovascular physiology, thermoregulation and skeletal muscle myopathy [7,8,9]. Yet the impact that alcohol ingestion has on exercise performance and more critically recovery has received less detailed scrutiny. This review aims to provide insights into the current knowledge around how alcohol acts to impair both exercise performance and the critical mechanisms by which alcohol acts at the cellular level to retard recovery following strenuous activity. 

## 2. Effect of Alcohol on Human Physiology

Alcohol consumption has a deleterious effect on a multitude of systems within the body and an in-depth analysis of each is beyond the scope of this review. However a brief insight into common symptoms linked to acute alcohol misuse will guide the following discussion on how alcohol influences human performance and recovery.

### 2.1. Skeletal Muscle

Multiple detrimental actions of alcohol within skeletal muscle are likely. Firstly, alcohol inhibits Ca^2+^ transients into the myocyte by inhibiting sarcolemmal Ca^2+^ channel actions. This action is reported in isolated human myotubes and rodent muscle tubes *in-vitro* [10,11,12]. Consequently this will impair excitation-contraction coupling, decreasing strength output. Yet human clinical data fails to support this *in-vitro* evidence [13]. Secondly, alcohol consumption may compromise sarcolemmal integrity, with evidence of greater plasma rises in the intracellular enzyme creatine kinase (CK), following alcohol ingestion and exercise [14]. Indeed, in rodents a supraphysiological dose of alcohol markedly increased plasma CK. Furthermore, in both electrically stimulated rodent muscles [15] and in human subjected to eccentric loading this not evident [13,16,17,18]. Thus clear mechanisms remain elusive, with a need for supporting clinical data. It is well understood that muscle cramps, pain and a loss of proprioception are common symptoms of alcohol misuse [19]; however the underlying mechanisms remain speculative.

### 2.2. Thermoregulation and Hydration

The effects of alcohol on hydration and its diuretic function are historically well recognised. The identification of alcohol as a potent diuretic date back to 1948, where a 10 mL excess urine production was evident following each gram of ethanol consumed [5]. The mechanism subsequently identified is the inhibition of anti-diuretic hormone (ADH) by ethanol [21], although this relationship is evident only in beverages containing greater than 4% (w/v) ethanol [21]. 

Alcohol has further been shown to act as a peripheral vasodilator. This presents several complications. Primarily this increases fluid loss through evaporation which further exacerbates the dehydration that is potentially already present. There is further an interference of central thermoregulatory mechanisms consequently resulting in a reduction in core body temperature [5,22]. Thus, not surprisingly, alcohol consumption has been repeatedly shown to decrease work tolerance in both high and low ambient temperatures [22,23,24].

### 2.3. Metabolism

In additional to being a readily accessible source of energy (29kJ per gram) [5], alcohol has a number of effects that bear ramifications for human metabolism. Alcohol-induced hypoglycemia has been proposed as a possible cause of symptoms common to alcohol misuse. The intake of high alcohol doses has been demonstrated to impair hepatic gluconeogenesis and subsequent glucose output [25,26], decrease the uptake of gluconeogenic precursors lactate and glycerol [27], and reduce muscle glycogen uptake and storage [27]. Alcohol has also been shown to induce a reactive hypoglycemia by exacerbating insulin secretion in the presence of a high carbohydrate meal [28]. While the detrimental effect of alcohol on glucose metabolism is strongly documented, some authors maintain that this can be negated if glycogen stores are maintained at homeostatic levels [25,29]. Specifically to exercise, acute alcohol intoxication inhibited the exercise induced rise in serum glucose concentration and caused a mild decrease in serum glucose during recovery from anaerobic exercise [25]. Further acute alcohol intoxication has been implicated in attenuating post exercise increases in serum fatty acid concentration [25]. These findings bear considerable ramifications for exercise performance and recovery. It is well documented in the literature that glucose availability plays a pivotal role in endurance performance [30,31] and further readily available stores of energy are necessary to fuel protein synthesis during muscle recovery from exercise [32].

### 2.4. Neurological

Alcohol is a well-known depressant and thus acts to reduce central nervous system (CNS) excitability and cerebral activity [33], as demonstrated by a slowed encephalographic rhythm [34]. Functionally alcohol has been repeatedly shown to exhibit a dose-dependent impairment of balance, reaction time, visual search, recognition, memory and accuracy of fine motor skills [1,9]. Variances in neurological activity have also been intricately linked to a disturbance in sleep length and quality with some authors observing a loss in sleep depth with a shorter time of rapid eye movement (REM) sleep and an increase in sleep at stage 1 [35,36].

The effect of alcohol on neurological function is likely to be caused by a myriad of factors. The aforementioned effect of alcohol on glucose metabolism could affect cerebral functioning leading to symptoms of alcohol intoxication [25]. Alternatively, the accumulation of acetaldehyde, a bi-product of alcohol metabolism, has been theorized as a potential cause of the aversive neurological symptoms associated with alcohol misuse [37]; however this prospect remains speculative [19]. Further the toxic effect of a group of substances collectively termed congeners often produced during the fermentation of alcohol, are likely to contribute to the reduction in CNS activity. Methanol, histamine and polyphenols are amongst the congeners best studied [38]. Serotonin regulation provides a further prospective mechanism as this hormone has both been shown to be increased in the presence of alcohol [38] and performs various cognitive functions including memory and learning.

## 3. Alcohol and Performance

Given the numerous and complex mechanisms by which ethanol impacts on physiological systems it can be strongly hypothesized that elevated blood alcohol concentrations at the time of exercise will impair performance. Remarkably there are relatively few clinical trials that address this question.

### 3.1. Aerobic Performance

Earlier studies found no significant consequence of alcohol on a sub-maximal endurance performance and a 5-mile treadmill time trial respectively [39,40]. Contrastingly and not surprisingly, there is also literature that demonstrates that alcohol is detrimental to endurance performance [41,42,43,44,45]. What is apparent is that a threshold exists at which point alcohol becomes detriment to aerobic performance. Cofan and colleagues describe an alcohol intoxication threshold of 20mmol/L of ethanol in both animal [12] and human [10] studies, beyond which did performance decrements become significant. Further research has elaborated that this cause-effect relationship may exist in a dose dependent manner [43]. 

### 3.2. Anaerobic Performance

Despite the long list of skeletal muscle and neurological symptomatology associated with alcohol consumption, the majority of literature has been unable to establish a significant cause-effect relationship between alcohol and anaerobic performance. To the reviewers knowledge McNaughton and Pierce [43] have conducted the only research that has identified an effect of alcohol on sprint performance. This research examined five sprinters using sprint time as a measure of performance and established a detrimental, albeit inconsistent, association between alcohol dosage and sprint performance. Alcohol was ingested immediately prior to exercise testing so this data is limited to the acute effects of alcohol intoxication and does not apply to more chronic hangover symptoms. Recent research has been unable to validate these findings, and have consistently seen no change in strength or power characteristics following acute alcohol ingestion [13,16,42]. Contrasting to McNaughton and Pierce, these studies have examined force output using an isokinetic dynamometer as their outcome measure of anaerobic performance. Comparatively time trial sprint performance incorporates a high degree of motor control and coordination and may provide insight as to why these findings cannot be replicated. 

## 4. Alcohol and Exercise Recovery

Most of the studies examining alcohol and athlete recovery have focused predominately on functional measures of muscle performance and blood borne markers of cellular tissue damage. To date, these studies have produced inconclusive results that fail to demonstrate a dose-dependency or critical threshold above which muscular recovery is compromised. Creatine kinase (CK) is an intra-muscular enzyme which when present in the peripheral circulation is widely used as a measure of muscle damage. Despite the clinical association between chronic alcohol abuse and skeletal muscle myopathy, acute ingestion appears to have little impact on exercise-mediated muscular damage [13,16,18]. The lack of results may be attributable to the parameters measured within these above mentioned trials. CK is highly variable and may not provide the best measure of muscle damage [45,48]. More recently, circulating levels of pro-inflammatory cytokines, released from the musculature may provide alternative measures of muscular stress and damage [47]. Inflammatory processes appear to be variably modulated by chronic and acute alcohol use. Prolonged alcoholism is associated with high circulating levels of pro-inflammatory mediators [49], whilst conversely acute consumption has been shown to decrease production of TNF-α and (IL-1) in rodent studies [50,51,52] It has yet to be established if cytokine concentrations are altered by acute alcohol ingestion during or immediately following intense exercise.

Similarly to the analysis of markers of muscle damage, the intra-muscular consequences of acute alcohol ingestion on aspects metabolic pathways of recovery are also ambiguous in humans. Alcohol ingestion immediately following prolonged cycling exercise has a modest impact to impair glycogen re-synthesis [48]. This action is dependent in part on alcohol replacing carbohydrates in energy-matched meals. Although acute suppression of glycogen synthesis may have been evident, examination of glycogen repletion over 24 hours demonstrated no long term detrimental impact of alcohol ingestion on muscle glycogen stores.

Of particular relevance to the recovery of strength athletes is the enhanced protein synthesis that occurs post-exercise to facilitate repair and adaptive hypertrophy [53]. Acute alcohol ingestion decreases muscle protein synthesis in a dose- and time-dependent manner, in the absence of an exercise stimulus. Alcohol facilitates this firstly by suppressing the phosphorylation and activation of the mTOR pathways, the critical kinase cascade regulating translation initiation [54]. Complementing the decreased activation of the protein synthetic pathway, alcohol increases the expression of muscle specific E3 ligases; atrogin-1 and Muscle-specific RING finger 1 (MuRF1) [55]. These proteins are up regulated by conditions that promote skeletal muscle atrophy. Interestingly this was not associated with increased proteolysis, suggesting alcohol primarily impairs protein synthesis. It remains to be confirmed in rodents subjected either to muscle loading or resistance exercise that alcohol impairs protein synthesis. Subsequent clinical data is also lacking and this remains a critical absence in the scientific literature.

Functionally, the consumption of moderate amounts of alcohol augments the loss of force associated strenuous eccentric exercise [18,56]. To the researchers knowledge Barnes, Mündel and Stannard have produced the only research that has used functional measures of muscle performance to identify an interaction between post-exercise muscle damage and alcohol [18,56]. This research established a significant decrease in average peak isometric, concentric and eccentric torques at 36 hours post-exercise. This decrement appeared to be exacerbated across all three variables in the group that consumed 1g per kg of body weight immediately post-exercise. Whilst this research provides new insights into the effect of alcohol consumption on post-exercise muscle recovery, further research is required to ascertain how this relationship exists and establish the physiological mechanisms governing this response.

## 5. Conclusion

Both the affects of alcohol on human physiology and the parameters that determine athletic performance are multi-factorial and extremely complicated. A significant body of literature has established an array of adverse symptoms caused by acute alcohol ingestion. However the notion that alcohol consumption effects performance has not received enough consistent validation to advance beyond being anecdotal. Nevertheless, just because alcohol is not yet comprehensively shown to have a negative influence on performance, does not imply this review advocates its use prior to, or following competition. Indeed, the data demonstrates a severe lack of analysis on the possible detrimental action of alcohol in the recovering athlete. However, based on the available experimental evidence in cellular and rodent-models, athletes should remain wary of ingesting alcohol following intense exercise, focusing instead on effective dietary strategies proven to enhance recovery.
